# Dysphagia in Alzheimer’s disease: a systematic review

**DOI:** 10.1590/1980-5764-DN-2021-0073

**Published:** 2022-05-23

**Authors:** Ana Mira, Rita Gonçalves, Inês Tello Rodrigues

**Affiliations:** 1 Escola Superior de Saúde do Alcoitão, Alcabideche, Portugal.; 2 Centro Hospitalar Universitário do Algarve, Centro de Medicina de Reabilitação do Sul, São Brás de Alportel, Portugal.; 3 Center for Innovative Care and Health Technology, Instituto Politécnico de Leiria, Leiria, Portugal.

**Keywords:** Deglutition Disorders, Alzheimer Disease, Disease Progression, Prevalence, Transtornos de Deglutição, Doença de Alzheimer, Progressão da Doença, Prevalência

## Abstract

Dysphagia is described as a highly relevant comorbidity of Alzheimer’s disease (AD). However, there is a scarcity of studies aiming at the characteristics and progression of dysphagia. Objective: The objective of this study was to identify the specific characteristics, progression, and prevalence of dysphagia in AD. Methods: Publications were searched in the PubMed (MEDLINE), EBSCO, ScienceDirect, and BASE databases. Critical appraisal and evidence-level analysis were conducted using the Joanna Briggs Institute and Effective Public Health Practice Project’s (EPHPP) tools. Results: A total of 26 studies were reviewed. Symptoms begin in the early stage of AD, as oral phase impairments, and progress to pharyngeal symptoms and swallowing apraxia in the later stages of AD. Dysphagia progresses, as AD, along a *continuum*, with severity depending on individual variability. There were no studies found on prevalence. Conclusions: Dysphagia is a complex and important comorbidity in AD that impacts the quality of life. No recent publications on prevalence may imply that is not being coded as a potential cause for pneumonia deaths in AD.

## RESUMO

A disfagia é uma comorbidade relevante da doença de Alzheimer (DA). No entanto, existem poucos estudos sobre as suas características e progressão. Objetivo: Identificar as características específicas, a progressão e a prevalência da disfagia na DA. Métodos: Pesquisa conduzida nas bases PubMed (*Medical Literature Analysis and Retrieval System Online* — MEDLINE), EBSCO, ScienceDirect e BASE. Avaliação crítica e análise do nível de evidência foram conduzidas usando as ferramentas do Joanna Briggs Institute e do Effective Public Health Practice Project (EPHPP). Resultados: Incluíram-se 26 estudos. Os sintomas iniciam-se no estádio inicial da DA, como alterações de fase oral, progredindo para alterações faríngeas e apraxia de deglutição no estádio grave. A disfagia progride, como a DA, num *continuum*, com a gravidade dependendo da variabilidade individual. Não foram encontrados estudos de prevalência. Conclusões: A disfagia é uma comorbidade complexa e importante que tem impacto na qualidade de vida. A escassez de publicações atuais de prevalência pode indicar que não é considerada como potencial causa de morte por pneumonia na DA.

## INTRODUCTION

Alzheimer’s disease (AD) was first described by Alois Alzheimer in 1907, which is a neurodegenerative disease^1^ that accounts for 60–70% of all cases of dementia^2^. Clinically, AD is characterized by behavioral and cognitive decline^1,3^ that typically results in symptoms originating from hippocampal and bilateral parietal-temporal dysfunction^2^. The progressive cognitive, behavioral, and neuropsychiatric symptoms have significant impacts on the affected individual’s autonomy^3^.

### Prevalence of dysphagia

The high prevalence of dysphagia among individuals with dementia is the result of age-related changes to sensory and motor functions, in addition to those produced by neuropathology^4^. The prevalence of dysphagia in moderate to severe AD is from 84 to 93%^5–7^. Ironically, dysphagia remains an overlooked symptom, even when its complications can lead to longer hospitalizations and increased health care costs^8^.

In AD, swallowing impairments are the leading cause for a progressive reduction in solid and liquid food intake^6^. Given that swallowing impairments directly affect food consumption, dysphagia may lead to weight loss, malnutrition, and dehydration^9–11^.

### Cortical deficits regarding dysphagia in Alzheimer’s disease

Cortical regions involved in normal swallowing are affected by AD, including the insula/inferior frontal gyrus, pars opercularis, anterior cingulate cortex, and anterior medial temporal lobe^12^. As AD progresses, individuals experience a significant deterioration in the swallowing mechanism^6^; although some studies report swallowing impairments in the early stages of AD, it is more pronounced in the later stages^9^.

Dysfunctions in cortical regions that control swallowing render the act of eating and drinking extremely effortful and may have devastating implications, such as increased risk for tracheal penetration and aspiration of foods, liquids, or even saliva, which can lead to aspiration pneumonia or death^10,12,13^. In AD, 70% of all deaths are related to pneumonia^14^.

### Dysphagia progression

In the early stages of AD, dysphagia undergoes a prolonged oral stage characterized by reduced lingual movement and delayed swallowing reflex^14,15^. This extended oral stage has been correlated with a longer duration for meal completion and, consequently, a risk of malnutrition^16^. The most frequent symptoms are oral residue after swallowing, mastication inefficacy, coughing or choking when consuming solid and/or liquid foods, and the need for verbal cues to initiate the swallowing reflex^17^. Some neurocognitive factors are associated with greater swallowing impairments, such as the inability to visually recognize foods, tactile and oral agnosia, and swallowing apraxia^18^.

Moderate AD stages are characterized by difficulties in bolus preparation, airway clearance, upper esophageal sphincter opening, and visible aspiration when conducting Fiberoptic Endoscopic Evaluation of Swallowing (FEES)^19^, where pharyngeal impairments can lead to aspiration before, during, or after swallowing^18^.

In the severe stage, swallowing difficulties are severe and significantly impinge on the individual’s quality of life^6,9^. At this stage, individuals with AD may experience swallowing apraxia^20^.

### Speech and Language Therapists’ role

Speech and language therapists (SLTs) have a fundamental role in the assessment and intervention of dysphagia, collaborating with diverse medical and nursing specialties in a variety of contexts^21,22^. Interventions by SLTs should be evidence-based and tailored to a unique set of difficulties of the person with dysphagia^23^.

The notable association between swallowing pattern, nutritional status, and general health status highlights the need for the specialized skills of SLTs in the effective management of dysphagia. Successful interventions help increase solid and liquid food intake, maintain nutritional status, and prevent morbidities such as pneumonia^10^.

The most frequent interventions used by SLTs are compensatory interventions (e.g., modification of diet consistency and/or postures), although their effects on the prevention of aspiration are variable^24^. The implementation of compensatory interventions is somehow related to the safety of oral food consumption, and their failure supports the use of acute alternative sources of nutrition^7^.

### Enteric nutrition

Enteric nutrition (percutaneous endoscopic gastrostomy [PEG] or nasogastric tube) in patients with AD or other dementias should only be administered in acute situations (e.g., cases of aspiration pneumonia or severe dysphagia). Generally, artificial nutrition yields no benefit on survival rates or decreasing the risk of aspiration in patients in the most advanced stages of dementia^18^.

### Aim

The primary aim of this review was to identify and describe the specific characteristics and symptom progression of dysphagia in AD in recent literature. The secondary aim was to investigate the available evidence on the prevalence of dysphagia in AD patients.

## METHODS

### Search strategy and selection criteria

In March 2020, two researchers independently conducted a search of publications between 2010 and 2020 by following a predefined protocol. This literature search was conducted on the PubMed, EBSCO, Science Direct, and BASE databases to identify studies on the characteristics of dysphagia in AD and its progression and prevalence. Reference lists of relevant articles were also reviewed. To ensure a thorough search, a protocol based on the PRISMA statement was designed, and combinations of search terms were determined (e.g., dysphagia, swallowing disorders, deglutition disorders, AD, prevalence, evolution, and progression). The inclusion criteria comprised peer-reviewed primary studies written in English, French, Spanish, or Portuguese published between 2010 and 2020.

### Critical appraisal and level of evidence

A critical appraisal and an evidence-level analysis were performed with the Joanna Briggs Institute Critical Appraisal Tools and the Effective Public Health Practice Project’s (EPHPP) “Quality Assessment Tool for Quantitative Studies.”

The Joanna Briggs Institute Critical Appraisal Tools^25^ were applied to each study according to its design and methodology. These tools allowed us to determine construct and internal validity, the sample establishment criteria, the risk of bias (in studies and by researchers), and the validity of the statistical tools chosen. Therefore, each critical appraisal tool allowed researchers to analyze the included studies independently. A consensus of critical appraisal was compiled in a table according to the study design. Of note, no study was excluded from the sample following the critical assessment.

Later, the process of assessing the level of evidence within the studies was conducted. To this end, the “Quality Assessment Tool for Quantitative Studies” tool, from the EPHPP, was used^26^. This tool analyzes the bias in the selection of the sample, study design, confounding variables, the knowledge of individuals regarding the objectives and/or procedures, methods of data collection, exclusions and/or withdrawals, integrity of the intervention, and analysis of the results; as a result, studies are assigned as possessing a strong, moderate, or weak level of evidence. The levels of evidence are attributed based on the application of the criteria listed in the tool’s appendix. The level assigned to each domain being studied is then reflected in the overall assessment of a given study. Three levels can be assigned: level 1 is “strong,” level 2 is “moderate,” and level 3 is “weak.” As no significant discrepancies were found in the application of the tool and in the levels of evidence established, a consensus on the level of evidence was reached.

### Data extraction and reporting

The data collected from the sample studies were organized into a table, which included author(s), publication year, study design, sample, objectives, data collection instruments, most pertinent results, and broader implications.

For the qualitative processing and synthesis of the studies, we used WebQDA software that identifies itself as a qualitative data analysis software.

## RESULTS

**Figure 1:**
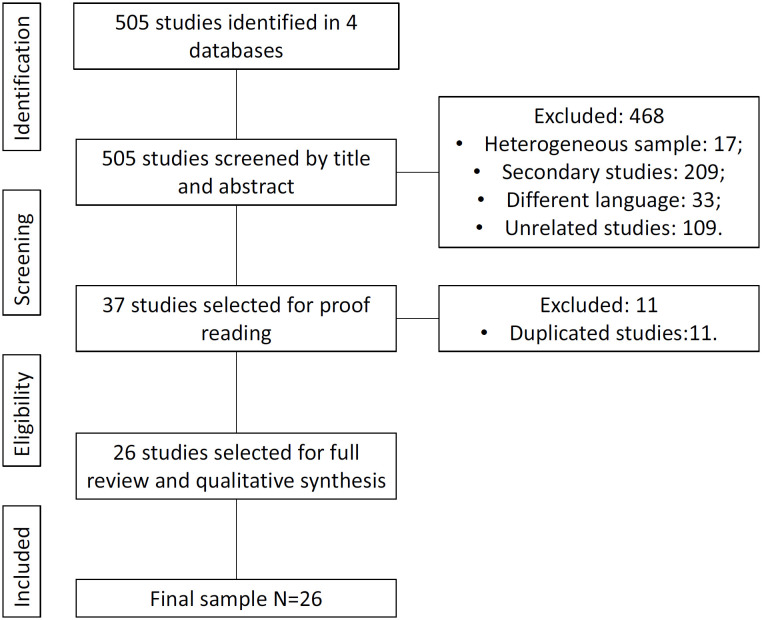
PRISMA flow diagram of the sampling process.

The initial search yielded 505 results from candidate studies that were screened by title and abstract. The screening process excluded 468 studies that failed to meet the inclusion criteria (e.g., heterogeneous sample, secondary or unrelated studies, studies in non-specified languages). After screening, both researchers proofread the remaining 37 studies and found that 11 of them were duplicated. Ultimately, 26 studies were included, as shown in Figure 1.

The level of evidence, which was determined by the EPHPP – Quality Assessment Tool for Quantitative Studies^26^, stated that the majority of the studies (69%) included had a moderate level of evidence.

With respect to study design, the final sample included 6 experimental studies and 20 observational studies, more specifically, non-randomized clinical trials (n=6), a case–control study (n=1), cohort studies (n=9), a case study (n=1), and longitudinal studies (n=9). The greatest limitations were found to be the study design, sample characteristics (e.g., number of participants, selection criteria, nonspecific dementia samples), and nonuniversal nomenclature used to describe the swallowing disorders.

**Figure 2:**
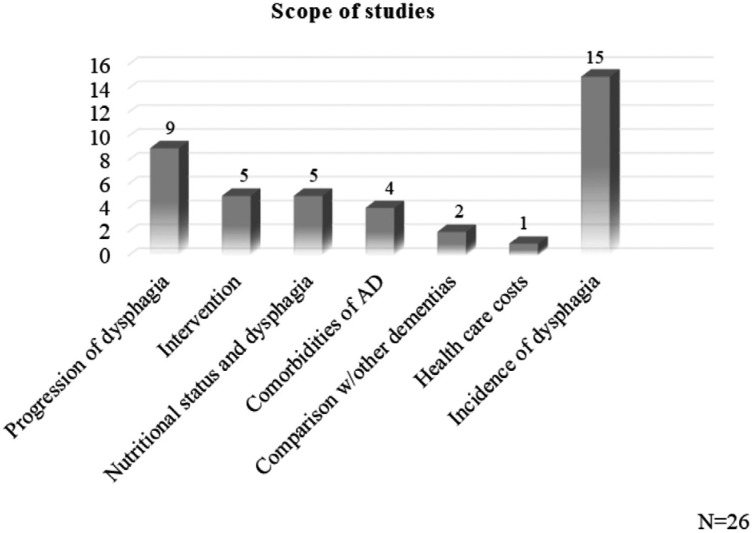
Scope of the sample studies.

Later, results were analyzed and synthesized, allowing to outline broad patterns and general characteristics. The scope of the studies is outlined in Figure 2.

**Figure 3:**
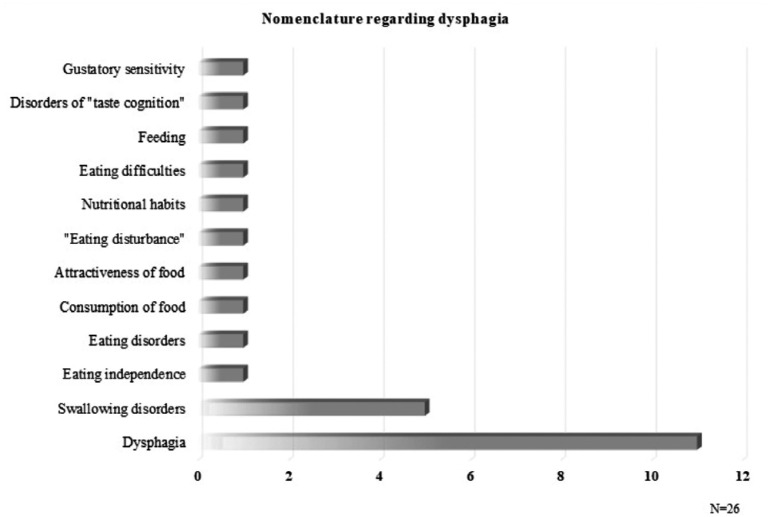
Nomenclature regarding dysphagia.

There were no epidemiological studies on prevalence published in the past 10 years, and the results presented a broad variety of topics and nomenclature regarding dysphagia in AD. Studies were found on the evolution of dysphagia, the correlation between nutritional status and dysphagia, intervention methodologies, health care-associated costs, comparison of dysphagia symptoms and progression between dementias, and dysphagia as a comorbidity of AD. The lack of consensus in nomenclature and criteria used for incidence and description of symptoms is shown in Figure 3.

Regarding dysphagia symptoms and their progression, stratification in stages was most commonly found according to the clinical dementia rating (CDR). A compilation of the most frequent symptoms is shown in Table 1.

**Table 1:** Dysphagia symptoms and progression according to clinical dementia rating.

Dysphagia symptoms	CDR1	CDR2	CDR3
Swallowing apraxia			x
Prolonged oral stage/phase	x	x	x
Reduced lingual movement	x	x	x
Mastication inefficacy/bolus preparation	x	x	x
Oral residue after swallowing	x	x	x
Delayed swallowing reflex	x	x	x
Coughing/airway clearance	x	x	x
Chocking	x	x	x
Upper esophageal sphincter opening		x	x
Visible aspiration (FEES)		x	x
Need for verbal cues to initiate swallow reflex		x	x
Oral agnosia			x

CDR: clinical dementia rating.

In the sample studies, the incidence of dysphagia varied from 2.4 to 100% (Table 2). The values shown were assessed by different methods and correspond to the AD population with different symptoms of dysphagia present in the samples of the studies included.

**Table 2:** Incidence of dysphagia in sample studies.

Year	Journal	Author(s)	Publication	Incidence of dysphagia (%) (samples)
2010	Arquivos Neuropsiquiatria	Correia et al^9^	Swallowing in moderate and severe phases of Alzheimer’s disease	27.8–71.9
2012	Geriatrics Gerontology International	Edahiro et al.^37^	Factors affecting independence in eating among elderly with Alzheimer’s disease	2.4–87.3
2013	European Psychiatry	Heun et al.^35^	Alzheimer’s disease and comorbidity: increased prevalence and possible risk factors of excess mortality in a naturalistic 7-year follow-up	11
	Alzheimer’s Disease Association Disorders	Tian et al.^36^	Health care utilization and costs among patients with AD with and without dysphagia	5.4
2014	Revista. Latino-Americana Enfermagem	Goes et al.^30^	Evaluation of dysphagia risk, nutritional status, and caloric intake in elderly patients with Alzheimer’s disease	86
	Geriatrics Gerontology International	Sato et al.^14^	Detecting signs of dysphagia in patients with Alzheimer’s disease with oral feeding in daily life	12.8–41
2015	Journal of Clinical Nursing	Chen et al.^28^	Effects of a feeding intervention in patients with Alzheimer’s disease and dysphagia	100
	PLoS ONE	Kai et al.^38^	Relationship between eating disturbance and dementia severity in patients with Alzheimer’s disease	81.4
	Turkish Journal of Medical Sciences	Yildiz et al.^34^	Malnutrition is associated with dementia severity and geriatric syndromes in patients with Alzheimer’s disease	5.4–36
2016	Journal of Nursing Home Research Sciences	Miranda et al ^31^	Undernutrition in institutionalized elderly patients with neurological diseases: comparison between different diagnostic criteria	63
	Clinical Neurophysiology	Seçil et al.^20^	Dysphagia in Alzheimer’s disease	75
	Medicine	Tang et al.^39^	Therapeutic efficacy of neuromuscular electrical stimulation and electromyographic biofeedback on Alzheimer’s disease patients with dysphagia	100
2018	Dementia and Neuropsychologia	Mastroianni and Forgerini^40^	Drug administration adjustments for elderly patients with dysphagia	100
2019	Journal of Parenteral and Enteral Nutrition	Ozsurekci et al.^32^	Timing of dysphagia screening in Alzheimer’s dementia	98.7
	Singapore Medical Journal	Shea et al.^41^	Chinese patients with Lewy body dementia had shorter survival and developed complications earlier than those with Alzheimer’s disease	12.9

Therefore, the sample studies were analyzed and synthesized according to their relevance in dysphagia understanding and management in the clinical setting. Relevant topics that were shown in recent literature were added to the study, analyzed, and described.

## DISCUSSION

The initial proposal for this study was to identify the specific characteristics of dysphagia in the different stages of AD. This was a goal rather challenging due to the design of the studies in recent literature. Designing and executing an experimental study in a target population with great individual variability as AD (clinical, neuropsychological, and cognitive-behavioral) may affect the quality and accuracy of study results. This may explain the abundance of studies in the literature with non-specified samples (i.e., dementia without any other specification), which were excluded from this study. The sample used in our own systematic review, which used observational (n=20) and experimental (n=6) studies, is not exempt from these challenges.

### Dysphagia characteristics and symptom progression

Dysphagia symptoms in early AD are centered around a longer oral phase with reduced lingual movement and delayed swallowing reflex. Understanding whether there would be functional changes in the cerebral cortex responsible for swallowing in the early stages of the disease prior to the onset of symptoms of oropharyngeal dysphagia was found in a study by Humbert and colleagues^12^. This study focused on the assessment of deficits in cortical control of swallowing in the early stages of AD and may have important clinical implications for educating patients with AD and their caregivers, early assessment, diagnosis, and intervention to minimize risks, future complications, and health care costs^12^.

The early-stage dysphagia symptoms have been correlated with a longer duration for meal completion and, consequently, a higher risk of malnutrition. The oral residue after swallowing, mastication inefficacy, coughing or choking when consuming solid and/or liquid foods, and the need for verbal cues to initiate the swallowing reflex are described in recent literature.

Dysphagia symptoms in moderate AD stages progress toward the pharyngeal phase where impairments can lead to aspiration before, during, or after swallowing. Difficulties in bolus preparation, airway clearance, upper esophageal sphincter opening, and visible aspiration when conducting FEES are the most common symptoms. Some neurocognitive factors are associated with greater swallowing impairments, such as the inability to visually recognize foods, tactile and oral agnosia, and swallowing apraxia.

The cortical deficits in the later stages of AD, oral, and pharyngeal phase difficulties were associated with difficulties in meal initiation, passivity, low attentional capacity, and refusal to eat. The decrease in speed and volume of intake relates to sensory-motor issues associated with cognitive changes resulting from AD^9^. In 2018, a study^27^ corroborated these results, focusing on the importance of manipulating the consistency of the foods offered to minimize the risk of aspiration and malnutrition^27^.

Swallowing difficulties in the later stage of AD are severe and greatly impair the quality of life. Also, patients with AD may experience swallowing apraxia at this stage.

A study by Seçil and colleagues in 2016^20^ found the changes in the electrophysiological parameters of swallowing in 75% of their sample, although no symptoms were shown. The authors also observed the following changes in swallowing as the disease progresses: subclinical dysphagia (early stage), dysphagia (moderate stage), and apraxia of swallowing (severe stage). The concept of progressive deterioration of the swallowing reflex appears in all studies.

Some studies have correlated dysphagia to the individual’s general nutritional status. For instance, studies^28–34^ found the correlations between nutritional aspects and dysphagia. Ultimately, the studies converge on the following results: the more severe the dysphagia, the worse the individual’s nutritional condition.

The evidence states unequivocally that AD comorbidities are interrelated^35^. The severity of dysphagia in the studies we sampled seems to directly influence nutrition, hydration, the presence of pressure ulcers, the presence and severity of respiratory infections, the severity of cognitive-behavioral changes, and the general health status of individuals with AD^34,35^.

### Prevalence and incidence of dysphagia in Alzheimer’s disease

The studies in this review allowed only to partially answer the research question. There were no studies found in the past 10 years regarding the prevalence of dysphagia in AD and the lack of consensus in nomenclature, and criteria used for incidence and description of symptoms were considerable limitations. Incidence data were classified and stratified according to CDR, others to the degree of severity of dysphagia, risk of dysphagia, or even risk of malnutrition. The authors used terms such as “malnutrition,” “low, moderate, high risk of dysphagia,” “eating disorders,” or “eating difficulties” that appear to indicate the incidence of swallowing disorders. Such nonspecific nomenclature is often used to describe issues associated with dysphagia, which has two potential explanations. First, clinicians are unable to determine where the physiological phenomenon of dysphagia begins, and the cognitive-behavioral phenomena associated with dementia end. The potential explanations provided suggest the presence of a vicious clinical circuit; the presence of AD disturbed intake, and the metabolic consequences worsening AD. Preclinical detection would be a valuable clinical goal that could influence early intervention, potentially slow down progression, and decrease health care cost. Second, the professional background of the researchers affects the nomenclature employed to describe the physiological phenomenon under study. The use of nonspecific nomenclature referring to swallowing disorders renders any generalizations dubious in validity. In addition, the frequency of a feature’s references does not necessarily represent the real prevalence of that feeding characteristic. This is due not only to the level of evidence of the studies and their methodological limitations, but also to the studies’ aims and to the instruments used to measure swallowing difficulties.

Regarding incidence, data were collected, analyzed, and summarized according to CDR. Incidence of dysphagia in the sample studies showed a major variation that ranged from 2.4 to 100%. The discrepancy of values could be explained by the differences in aim, samples, and assessment methods, and correspond to the AD population with different symptoms in the samples of the studies included. Researchers should aim for a consensual nomenclature (that does not depend on the researcher’s professional background) as well as homogeneous samples in their studies. Therefore, a qualitative analysis of the most relevant topics in recent literature should be conducted with clinical implications in managing dysphagia in AD.

## Further discussion

A study by Tian and colleagues^36^ proved to be unique in the literature; they retrospectively studied two databases of North America’s health care subsystems within a 4-year period and found that patients with AD and dysphagia use health services significantly more and at higher costs. This corroborates the idea that early intervention in dysphagia in AD may help to lower health care-related expenditures. Further research in this area could spur political and clinical decision-makers to fund early intervention.

### Limitations

The main proposal of this study was to present a global and largely inclusive perspective on swallowing disorders in AD patients.

An effort was made to systematize the information into main categories; however, it might not have involved a consensual approach.

Several limitations should be borne in mind when interpreting our results. Selection bias may be present. To increase data collection, the identification of grey (unpublished) literature could offer new insights, although it is also a controversial procedure due to its unconventional format and a lack of a peer-reviewed process.

Another important source of bias in results comparison is the variety of the studies included. For example, there were a limited number of experimental studies and a significant number of observational studies, variation of protocols and outcome measures, and no assessment of the risk of bias. Furthermore, studies evaluating interventions and management were unable to blind direct care providers due to the nature of the intervention. The diversity of terms regarding dysphagia with a lack of consensus between authors was also a serious limitation to the data analysis, increasing the risk of bias.

### Future directions

Although there is evidence in the literature that dysphagia is an important symptom in AD, no studies in the past decade were found on its prevalence or variations in prevalence as a function of disease progression. Future research is encouraged to focus on prevalence so that clinicians, politicians, and the public at large can be better informed about dysphagia, and better diagnostic tools can be developed.

Only a handful of studies showed that changes in the cortical swallowing network occur early in AD and may be correlated with early functional changes in swallowing; however, the neuropathophysiology of dysphagia in AD remains unclear. Therefore, future studies should focus on the neuropathophysiology of swallowing impairments in AD so that the association with functional changes and symptoms is brought to light.

Dysphagia presents itself as one of the most impactful comorbidities of AD, yet its supporting evidence is scarce. Although the literature indicates that dysphagia affects both the oral and pharyngeal stages of swallowing in AD, no studies were found on the esophageal stage of swallowing. Future research on the specific symptoms of each stage of swallowing in AD would improve assessment and intervention, as well as the quality of life.

The research team also found that most studies differ in their nomenclature on dysphagia, a problem that urgently needs a solution.

In addition, different methodologies were used to examine dysphagia in AD, with no specific and individualized swallowing assessment being consistently used. Also, few studies examined specific interventions for dysphagia in individuals with AD. Postural adjustments, food consistency modifications, electric or sensory stimulation, and motor training as intervention have been thoroughly studied in several neurodegenerative diseases, and future research should aim for the same standard to be applied in dysphagia interventions in AD.

In conclusion, dysphagia in AD is described in the literature as an important comorbidity due to its impact on the quality of life of individuals. It has a complex, multifaceted, and variable clinical presentation. Dysphagia, such as AD, progresses along a continuum of symptoms that decrease the individual’s quality of life and increase the health care costs.

Long before symptoms appear, there are cortical changes in the neural networks responsible for swallowing. Dysphagia is linked to other comorbidities of AD unequivocally: more severe dysphagia leads to severe malnutrition and dehydration, severe respiratory infections, falls, pressure ulcers, cognitive-behavioral decline, and even the individual’s death. This systematic review aims to impact clinicians in the assessment and diagnosis of dysphagia in AD and in the design of a specific and individualized therapeutic program that aims to prevent future clinical complications or even death.

Regarding the prevalence of dysphagia in AD, there are no epidemiological studies on the prevalence of this comorbidity in the literature in the past 10 years but only estimated data (with wide prevalence windows) from international health associations that (specifically) study the pathology and the comorbidities associated with it. It is considered imperative to understand the real prevalence of dysphagia in AD so that policymakers and clinicians can converge toward early intervention and reducing the burden on health systems. No up-to-date study on the prevalence of dysphagia in AD could also mean that this comorbidity could be underdiagnosed and not noted as a leading cause of death from pneumonia in patients with AD.

As a partial answer to the research question, it is concluded that dysphagia is undeniably presented as an important, impactful, continuous disorder, often associated with the progression of AD. It evolves, like the disease, in a degenerative sense and contributes to the progressive decrease in the individual’s quality of life and to the increase in access and associated costs in health (medication, hospitalization, among others).

The lack of a clear nomenclature, incidence data, and recent prevalence studies contributes to lessen the quality of dysphagia understanding and management in AD.

This review contributes to a global view of the phenomenon of dysphagia in AD and serves as a basis for future research.
